# Supplementary material for: Computer-Aided Designing Peptide Inhibitors of Human Hematopoietic Prostaglandin D2 Synthase Combined Molecular Docking and Molecular Dynamics Simulation
Source: Molecules. 2023 Aug 7;28(15):5933. doi: 10.3390/molecules28155933 (PMC10421073; doi:10.3390/molecules28155933)
Supplement: Supplementary file 1 [file molecules-28-05933-s001.zip › Supplemented File S1.pdf]

Command line for Rosetta and Gromacs

##Rosetta command line for protein Relax
##First round

```
relax.mpi.linuxgccrelease -s input_pdb.pdb -relax:constrain_relax_to_start_coords -ramp_constraints false -
relax:coord_constrain_sidechains -nstruct 50 -ex1 -ex2 -use_input_sc -flip_HNQ -no_optH false
```

##Second round

```
relax.mpi.linuxgccrelease -s 1.pdb -nstruct 50 -ex1 -ex2 -use_input_sc -flip_HNQ -no_optH false
-relax:cartesian -score:weights ref2015_cart -crystal_refine
```

##Python script for generating peptides

```
import random
import os
def seq_gen(generate_number=3,mers=5):

    residues_list = ['A', 'G', 'D', 'N', 'F', 'P', 'Q', 'C', 'E', 'H', 'I', 'K', 'L', 'M', 'R', 'S', 'T', 'V', 'W', 'Y']
    for i in range(generate_number):
        a =(random.choices(residues_list,k=mers))
        seq = ''
        seq = seq.join(a)

        if not os.path.exists('%s'%seq):
            os.mkdir('%s'%seq)
        f = open('%s/buildpep.sh'%seq,'w')
        f.write('BuildPeptide.mpi.linuxgccrelease -in:file:fasta %s/1.fasta -out:file:o %s/1.pdb'%(seq,seq))
        f.close()
        f = open('%s/1.fasta'%seq,'w')
        f.write('%s'%seq)
        f.close()
        os.system('bash %s/buildpep.sh'%seq)

seq_gen()
```

##Accommodating peptide into receptor and performing docking

```
import os
path = os.getcwd() + '/1'
pep = os.listdir(path)
list= []
for i in pep:
    if len(i)>2:
        list.append(i)
for i in list:
    os.system('cp 1.py 1.sh editconf.sh protein.pdb run.xml %s/%s'%(path,i))
cpu_cores = 70
def multi_run(cores=cpu_cores):
    a = int(len(list)/cores)

    for i in range(cores):
        for k in range(a+1):
            try:
                f = open('run%s.sh'%str(i),'a+')
                f.write('\ncd %s/%s\n'%(path,list[k+i*(a+1)]))
                f.write('bash editconf.sh')
                f.close()
            except:
                pass
multi_run()
f=open('run.sh','a+')
for i in range(cpu_cores):
    f.write('nohup bash run%s.sh &\n' % str(i))
f.close()
```

##Rosetta command line for peptide conformation optimization

```
rosetta_scripts.mpi.linuxgccrelease -s 1.pdb -parser:protocol run.xml -in:file:fullatom -ignore_unrecognized_res -ex1 -ex2 -
overwrite -restore_talaris_behavior
```

##Gromacs command line for peptide conformation optimization

```
gmx_mpi editconf -f 1.pdb -center coord_x coord_y coord_z -o ligo.pdb
```

##Python script for renaming protein chain

```
import sys
pdbdata = open('ligo.pdb','r').readlines()
chain = 'B'
output = open('ligo.pdb','w')
for line in pdbdata:
```

```
        if line[0:4] =='ATOM':
            #print(line.replace('\n',''))
            try:
                newline = '%s%s%s'%(line[0:21],chain,line[22:])
                output.write(newline)
            except:
                print('bad')
                #output.write(line)
```

##Rosetta script for peptide conformation optimization

```
<ROSETTASCRIPTS>
  <SCOREFXNS>
    <ScoreFunction name="r2014" weights="talaris2014_cart"/>
  </SCOREFXNS>

  <RESIDUE_SELECTORS>
  </RESIDUE_SELECTORS>
  <TASKOPERATIONS>
  </TASKOPERATIONS>

  <FILTERS>
  </FILTERS>

  <MOVERS>
    <MinMover name="minimize" scorefxn="r2014" chi="1" bb="1"    tolerance="0.0000000001" max_iter="1000000" />
    <CartesianMD    name="MD" scorefxn="r2014" nstep="100000" temp="200"    premin="500" />
  </MOVERS>
  <APPLY_TO_POSE>
  </APPLY_TO_POSE>
  <PROTOCOLS>
    <Add mover_name="minimize"/>
    <Add mover_name="MD"/>
  </PROTOCOLS>
  <OUTPUT />
</ROSETTASCRIPTS>
```

##Rosetta command line for molecular docking

```
rosetta_scripts.mpi.linuxgccrelease -s 1.pdb -parser:protocol pep_refine.xml -in:file:fullatom -ignore_unrecognized_res -ex1
-ex2 –overwrite –nstruct 100
```

##Rosetta script for molecular docking

```
<ROSETTASCRIPTS>
    <SCOREFXNS>
        <ScoreFunction name="r2015" weights="ref2015" />
    </SCOREFXNS>

    <RESIDUE_SELECTORS>
    </RESIDUE_SELECTORS>
    <TASKOPERATIONS>
        <RestrictToRepacking name="no_design" />
    </TASKOPERATIONS>
    <FILTERS>
    </FILTERS>

    <MOVERS>
        <FlexPepDock name="dock" pep_refine="1"    />
        <InterfaceAnalyzerMover name="dg" scorefxn="r2015" packstat="1" pack_input="0" jump="1" tracer="0"
use_jobname="1" resfile="0" />
        <FastRelax name="fastrelax" scorefxn="r2015" task_operations="no_design"
relaxscript="InterfaceRelax2019" >
            <MoveMap name="only_move_protein" >
                <Chain number="1" chi="1" bb="1" />
            </MoveMap>
        </FastRelax>
        <MinMover name="min_mover" scorefxn="r2015" tolerance="0.000001" bb="1" chi="1" jump="1"
max_iter="5000" />
    </MOVERS>
    <APPLY_TO_POSE>
    </APPLY_TO_POSE>
    <PROTOCOLS>
        <Add mover="dock" />
        <Add mover="min_mover"/>
        <Add mover="fastrelax" />
        <Add mover="dg" />
    </PROTOCOLS>
```

```
        <OUTPUT />
</ROSETTASCRIPTS>
```

Step by step validating Rosetta script:

Step 1: Accommodating peptide into the active center of protein receptor:

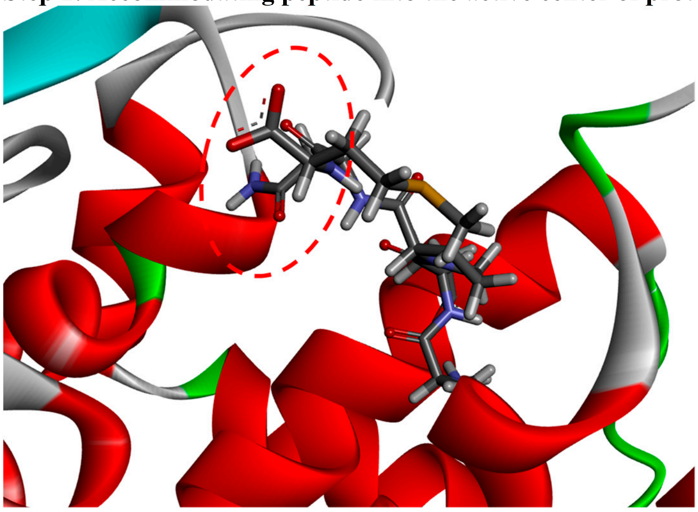

Step 2: Rosetta script only contain FlexPepDock to perform peptide refine:

The script and command line as below:

```
<ROSETTASCRIPTS>
  <SCOREFXNS>
    <ScoreFunction name="r2015" weights="ref2015" />
  </SCOREFXNS>

  <RESIDUE_SELECTORS>
  </RESIDUE_SELECTORS>
  <TASKOPERATIONS>
    <RestrictToRepacking name="no_design" />
  </TASKOPERATIONS>
  <FILTERS>
  </FILTERS>

  <MOVERS>
    <FlexPepDock name="dock" pep_refine="1"  />
    <InterfaceAnalyzerMover name="dg" scorefxn="r2015" packstat="1" pack_input="0" jump="1" tracer="0" use_jobname="1" resfile="0" />
    <FastRelax name="fastrelax" scorefxn="r2015" task_operations="no_design" relaxscript="InterfaceRelax2019" >
          <MoveMap name="only_move_protein" >
            <Chain number="1" chi="1" bb="1" />
          </MoveMap>
        </FastRelax>
        <MinMover name="min_mover" scorefxn="r2015" tolerance="0.000001" bb="1" chi="1" jump="1" max_iter="5000" />
  </MOVERS>
  <APPLY_TO_POSE>
  </APPLY_TO_POSE>
  <PROTOCOLS>
    <Add mover="dock" />
  </PROTOCOLS>
  <OUTPUT />
</ROSETTASCRIPTS>
```

```
rosetta_scripts.mpi.linuxgccrelease -s 1.pdb -parser:protocol pep_refine.xml -in:file:fullatom -ignore_unrecognized_res -ex1 -ex2 -overwrite
```

Resulted refined peptide steric location:

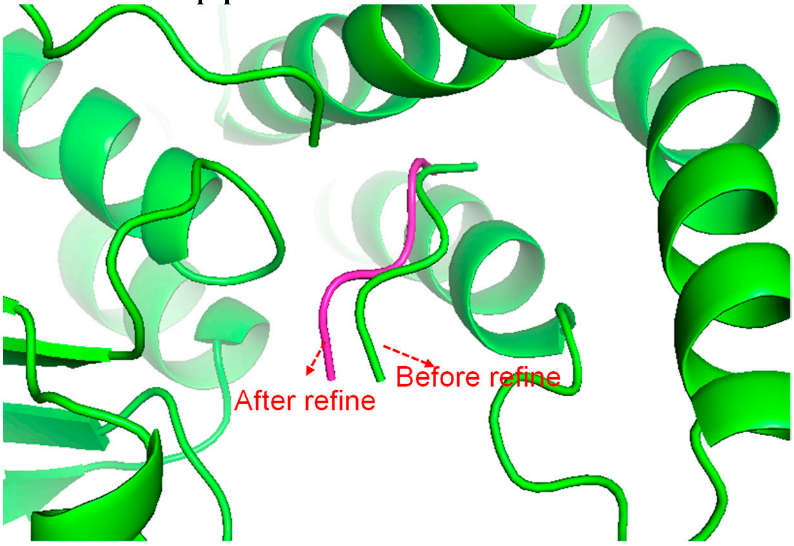

Step 3: Rosetta script only contain Minmover to perform whole structure refine:

The script and command line as below:

```
<ROSETTASCRIPTS>
  <SCOREFXNS>
    <ScoreFunction name="r2015" weights="ref2015" />
  </SCOREFXNS>

  <RESIDUE_SELECTORS>
  </RESIDUE_SELECTORS>
  <TASKOPERATIONS>
    <RestrictToRepacking name="no_design" />
  </TASKOPERATIONS>
  <FILTERS>
  </FILTERS>

  <MOVERS>
    <FlexPepDock name="dock" pep_refine="1"  />
    <InterfaceAnalyzerMover name="dg" scorefxn="r2015" packstat="1" pack_input="0" jump="1" tracer="0" use_jobname="1" resfile="0" />
    <FastRelax name="fastrelax" scorefxn="r2015" task_operations="no_design" relaxscript="InterfaceRelax2019" >
          <MoveMap name="only_move_protein" >
            <Chain number="1" chi="1" bb="1" />
          </MoveMap>
        </FastRelax>
        <MinMover name="min_mover" scorefxn="r2015" tolerance="0.000001" bb="1" chi="1" jump="1" max_iter="5000" />
  </MOVERS>
  <APPLY_TO_POSE>
  </APPLY_TO_POSE>
  <PROTOCOLS>

    <Add mover="min_mover"/>

  </PROTOCOLS>
  <OUTPUT />
</ROSETTASCRIPTS>
```

```
rosetta_scripts.mpi.linuxgccrelease -s 1.pdb -parser:protocol pep_refine.xml -in:file:fullatom -ignore_unrecognized_res -ex1 -ex2 -overwrite
```

Resulted overall structure compared with native structure:

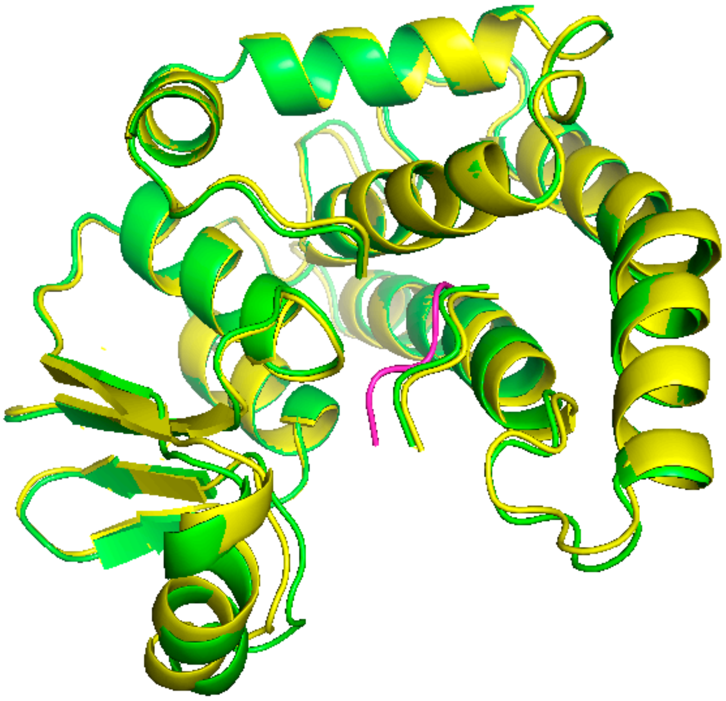

Protein receptor without Minmover for structural refine in green, protein and peptide after Minmover refine in yellow, RMSD = 0.455 Å

Step 4: Rosetta script only contain Fastrelax to perform whole structure refine and repack:

The script and command line as below:

```
<ROSETTASCRIPTS>
  <SCOREFXNS>
    <ScoreFunction name="r2015" weights="ref2015" />
  </SCOREFXNS>

  <RESIDUE_SELECTORS>
  </RESIDUE_SELECTORS>
  <TASKOPERATIONS>
    <RestrictToRepacking name="no_design" />
  </TASKOPERATIONS>
  <FILTERS>
  </FILTERS>

  <MOVERS>
    <FlexPepDock name="dock" pep_refine="1"  />
    <InterfaceAnalyzerMover name="dg" scorefxn="r2015" packstat="1" pack_input="0" jump="1" tracer="0" use_jobname="1" resfile="0" />
    <FastRelax name="fastrelax" scorefxn="r2015" task_operations="no_design" relaxscript="InterfaceRelax2019" >
          <MoveMap name="only_move_protein" >
            <Chain number="1" chi="1" bb="1" />
          </MoveMap>
        </FastRelax>
        <MinMover name="min_mover" scorefxn="r2015" tolerance="0.000001" bb="1" chi="1" jump="1" max_iter="5000" />
  </MOVERS>
  <APPLY_TO_POSE>
  </APPLY_TO_POSE>
  <PROTOCOLS>

    <Add mover="fastrelax" />

  </PROTOCOLS>
  <OUTPUT />
</ROSETTASCRIPTS>
```

```
rosetta_scripts.mpi.linuxgccrelease -s 1.pdb -parser:protocol pep_refine.xml -in:file:fullatom -ignore_unrecognized_res -ex1 -ex2 -overwrite
```

Resulted overall structure compared with native structure:

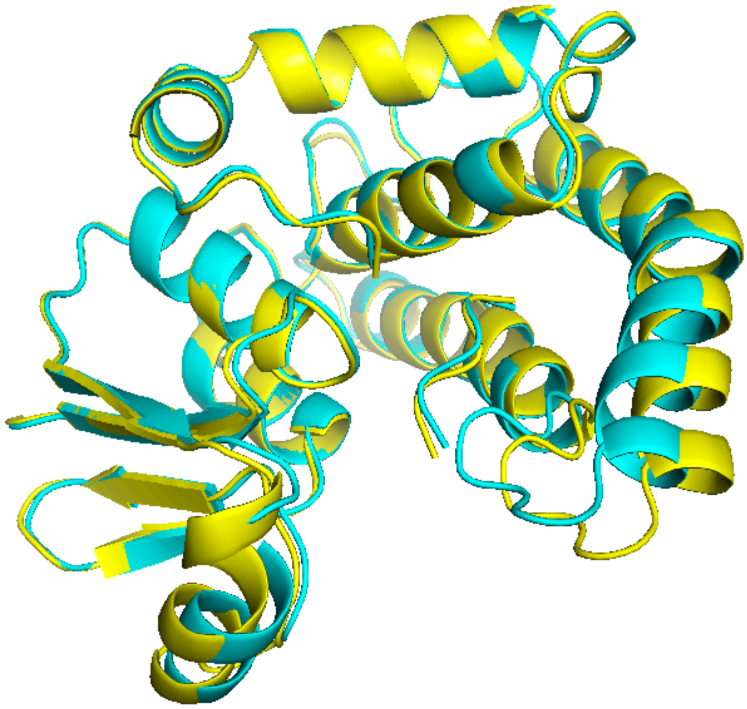

Overall structure without Fastrelax in yellow, overall structure after Fastrelax refine in cyan, RMSD = 0.566 Å
